# The effects of physical activity on diabetic retinopathy in type 2 diabetes using automated vascular analysis: a cohort study

**DOI:** 10.7189/jogh.15.04319

**Published:** 2025-12-05

**Authors:** Zhaoyu Xiang, Senlin Lin, Yi Xu, Lina Lu, Yan Shi, Yuheng Wang, Qinping Yang, Saiguang Ling, Dengji Zhou, Xinran Qin, Minna Cheng, Haidong Zou, Yingyan Ma

**Affiliations:** 1 Shanghai Eye Diseases Prevention &Treatment Center/Shanghai Eye Hospital, School of Medicine, Tongji University, Shanghai, China; 2 Eye Public Health Research Center, School of Medicine, Tongji University, Shanghai, China; 3 Department of Ophthalmology, Shanghai General Hospital, School of Medicine, Shanghai Jiao Tong University, Shanghai, China; 4 Shanghai Engineering Center for Precise Diagnosis and Treatment of Eye Diseases, Shanghai, China; 5 National Clinical Research Center for Eye Diseases, Shanghai, China; 6 Division of Noncommunicable Diseases and Injury, Shanghai Municipal Center for Disease Control and Prevention, Shanghai, China; 7 National Clinical Research Center for Aging and Medicine, Huashan Hospital, Fudan University, Shanghai, China; 8 EVision Technology (Beijing) Co., Ltd., Shangdi Information Industry Base, Beijing, China

## Abstract

**Background:**

Evidence regarding the association between physical activity (PA) and diabetic retinopathy (DR) remains inconsistent. Furthermore, its effects on retinal vessel diameters in type 2 diabetes are not well established. We aimed to investigate the relationship between PA, DR, and retinal vessel diameters, explore underlying mechanisms, and identify protective exercise regimens.

**Methods:**

We included patients with type 2 diabetes from the Shanghai Cohort Study of Diabetic Eye Disease. Retinal vessel diameters were measured using computer vision and deep learning. Anthropometric data were collected using standard methods, and PA data through interviews. In 2017, participants were categorised by their DR status. Those without DR were divided into active and inactive groups and followed for three years to assess the effect of PA. For statistical analyses, we used independent t*-*tests, χ^2^ tests, one-way analysis of variance, Bonferroni tests, multiple linear and logistic regression models, Kaplan-Meier, and Cox regression models.

**Results:**

In the cross-sectional analysis, we analysed a sample of 42 992 individuals, with a mean age of 64.42 (standard deviation (SD) = 6.87) years. PA was associated with reduced odds of moderate and severe non-proliferative DR, and with wider retinal arterioles and venules. In the longitudinal cohort, we analysed 3669 individuals, with a mean age of 63.1 (SD = 6.65) years. PA was a protective factor against incident DR (hazard ratio = 0.812; 95% confidence interval = 0.679–0.971) and was associated with increased peripheral retinal arteriolar calibre and arterio-venous ratio.

**Conclusions:**

PA improved retinal vessel diameters and lowered DR incidence, highlighting the necessity for further research into the physiological mechanisms linking PA and DR. Promoting awareness and engagement in moderate/high-intensity exercise may enhance diabetes health management.

**Registration:**

ClinicalTrials.gov NCT03665090.

The American Diabetes Association recommends that adults with diabetes engage in ≥150-minute of moderate-intensity exercise or ≥75-minute of vigorous-intensity exercise weekly, distributed over at least three days [1]. Physical activity (PA) protects patients with diabetes from macrovascular complications and reduces mortality, while also improving glycated haemoglobin, triglycerides, blood pressure, and insulin resistance [2].

Diabetic retinopathy (DR), a common microvascular complication of diabetes, exhibits inconsistent associations with PA (Table S1 in the **Online Supplementary Document**). Cross-sectional studies provide mixed evidence; while the German Gutenberg Health Study and others report that regular PA is associated with reduced DR prevalence [3,4], several large-scale studies report no significant association between PA and DR [5,6]. Longitudinal studies also reveal inconsistencies; early type 1 diabetes cohorts detected no PA-DR associations [7,8], whereas subsequent research identified protective effects against DR progression in both type 1 and type 2 diabetes [9–12]. Methodological discrepancies in outcome definitions, PA assessment, and sample sizes likely contribute to these inconsistencies. Current evidence suggests PA is non-detrimental to DR and may be protective in specific contexts. We analysed data from the Shanghai Cohort Study of Diabetic Eye Disease (SCODE) (2017–20) to identify protective exercise regimens and employed two-sample Mendelian randomisation (MR) to address confounders.

PA may influence DR by affecting retinal vessels. In healthy individuals, PA increases the central retinal artery equivalent (CRAE), reduces the central retinal vein equivalent (CRVE), and improves the arteriole-to-venule ratio (AVR) [13], a recognised independent cardiovascular risk marker [14]. Retinal microvascular damage may precede clinical DR [15]. Although PA improves retinal vascular parameters in high cardiovascular risk patients [16], its effects in diabetic patients, especially longitudinally, remain understudied. We investigated the relationship between leisure-time PA and retinal vessel diameters in patients with type 2 diabetes from the SCODE to explore the links among PA, retinal vessel diameters, and DR, providing a clinical foundation for understanding protective mechanisms of PA.

## METHODS

### Recruitment

We selected patients with type 2 diabetes from the SCODE cohort (2017–20). All participants provided written informed consent.

**Figure 1:**
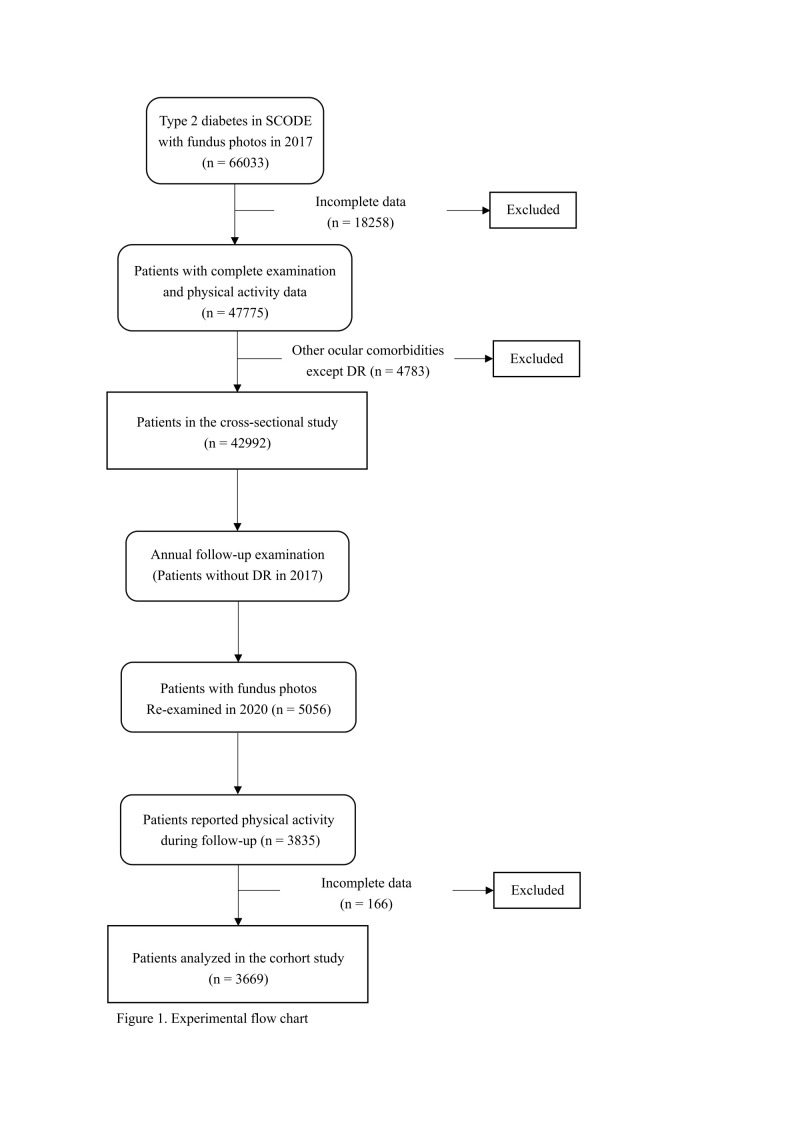
Flowchart of participant selection for cross-sectional and cohort analyses.

In 2017, fundus photographs, PA data, and anthropometric data were collected. After excluding 4783 patients with ocular comorbidities other than DR, 42 992 patients remained for cross-sectional analysis. The inclusion and exclusion criteria have been previously described [17]. From 2017 to 2020, 5056 patients without DR at baseline were followed annually, with fundus photographs taken in 2020; 3669 with consistent PA and complete data were included in the cohort analysis (**Figure 1**). We followed the STROBE guidelines (Checklist S1 in the **Online Supplementary Document**) [18]. We used baseline (2017) and three-year average data (2017–20).

### Anthropometry, PA, fundus photographs, and retinal vessel diameter measurements

We obtained anthropometric measurements (including weight, height, body mass index, blood pressure, fasting blood glucose, and glycated haemoglobin), PA data, and retinal vessel diameters using standardised procedures (Text S1 in the **Online Supplementary Document**) [17]. Physicians recorded the intensity, frequency, and duration of the primary leisure-time PA performed each week. We obtained retinal photographs using a 45° 3.78-megapixel digital camera (Topcon NW400, Tokyo, Japan). Physicians diagnosed DR based on macular fundus photography according to the International Clinical Classification of DR [19]. We calculated CRAE, CRVE, and AVR (AVR = CRAE/CRVE) using the modified Parr-Hubbard formula [20]. Intraclass correlation coefficients were 0.860 for CRAE, 0.805 for CRVE, and 0.807 for AVR.

### Two-sample MR analysis

Adhering to STROBE-MR guidelines, we used data for ‘duration of moderate activity’ (GWAS ID: ukb-a-509), curated by the Neale Laboratory from the IEU OpenGWAS project, as the exposure. We obtained the outcome data for DR from the FinnGen consortium (finngen_R12_DM_RETINOPATHY_EXMORE). For the primary analysis, we used the inverse-variance weighted (IVW) method, complemented by MR-Egger regression and the weighted median methods. The IVW method treats each genetic variant (single-nucleotide polymorphism (SNP)) as an independent natural experiment, combining the SNP-outcome estimates using a precision-weighted meta-analysis. The estimated odds ratio represents the change in DR risk per standard deviation (SD) increase in duration of moderate activity. We implemented rigorous quality control to satisfy the three core MR assumptions: that the instrumental variable (IV) is associated with exposure, that IV affects outcomes only through exposure, and that IV is not associated with confounders.

These included selecting genome-wide significant SNPs (*P* < 5 × 10^−6^), performing linkage disequilibrium clumping (r^2 ^< 0.001 within 10 000 kb window) using the 1000 Genomes Project European reference panel, screening for confounding factors and pleiotropy via LDlink (r^2 ^< 0.1 within 500 000 b window) [21], and excluding weak IV (F > 10; F = *β*^2^/SE^2^).

We assessed heterogeneity using Cochran’s Q test (*P* > 0.05 indicating homogeneity). Sensitivity analyses included MR-Egger regression to assess directional pleiotropy (*P* > 0.05 indicating no evidence), and MR-PRESSO to detect and correct for horizontal pleiotropy (removing outliers if *P* < 0.05).

For reverse MR analysis, we swapped the exposure and outcome data sets relative to the forward analysis, while keeping the analytical procedures identical. Reverse MR analysis excluded reverse causation (*P*_IVW _> 0.05, indicating no reverse association).

Additionally, we performed MR analysis using ‘duration of vigorous activity’ (GWAS ID: ukb-a-512) as the exposure to explore the effect of exercise intensity on DR, applying identical analytical procedures.

### Statistical analysis

We used SPSS, version 22.0 (IBM Corp., Armonk, New York, USA), with a significance threshold of *P* < 0.05. We presented continuous variables as mean (SD) and categorical variables as numbers (percentages). Due to a significant correlation between left and right eye data (*P* < 0.05), we only analysed right-eye data.

We used independent t-tests to compare continuous variables and χ^2^ tests to compare categorical variables between groups. We used one-way analysis of variance to assess between-group differences, with the Bonferroni correction for *post-hoc* comparisons. We used multiple linear regression to identify factors associated with retinal vessel diameters, and logistic regression to assess associations between various factors and the prevalence of DR, non-proliferative DR (NPDR), and proliferative DR (PDR). We used the Kaplan-Meier test to compare DR-free survival between groups. Additionally, we used a Cox regression model to identify factors associated with DR incidence. The inclusion criterion for independent variables in the regression equation was *P* ≤ 0.05, while the exclusion criterion was *P* ≥ 0.10. We coded sex as male = 1 and female = 0. For MR analyses, we used *R*, version 4.4.3 (R Core Team, Vienna, Austria) with TwoSampleMR, MRPRESSO, ieugwasr, and plinkbinr packages, using *P*_IVW_<0.05 as a significance threshold.

## RESULTS

### Participants in the cross-sectional analysis

The mean age of the 42 992 patients in 2017 was 64.42 (SD = 6.87) years, with a mean diabetes duration of 8.25 (SD = 5.49) years. Approximately 20.19% patients had DR, with 6.5% having mild, 11.6% moderate, and 1.7% severe NPDR, while 0.3% had PDR (Table S2 in the **Online Supplementary Document**).

We applied a stepwise logistic regression to analyse the risk factors for DR. The independent variables included age, sex, duration of diabetes, glycaemic control, control of blood pressure, insulin, smoking, drinking, AVR, peripheral AVR (PAVR), and active PA (Table S3 in the **Online Supplementary Document**). The results showed that patients with DR were younger, more likely to be female, had longer duration of diabetes, poorer glycaemic control, poorer blood pressure control, more insulin use, smaller AVR, and smaller PAVR compared to those without DR.

### Association of PA with the prevalence of DR

Univariate analysis revealed that PA was not significantly associated with the prevalence of DR (P = 0.20) or PDR (*P* = 0.18) but was significantly associated with the NPDR severity (χ^2^ = 62.92; *P* = 1.14 × 10^−14^) (Table S4 in the **Online Supplementary Document**). After multivariate adjustment (age, sex, duration of diabetes, glycaemic control, control of blood pressure, insulin, smoking, drinking, AVR, and PAVR), PA was a protective factor against moderate and severe NPDR.

Analysis of exercise components revealed that, after multivariate adjustment, performing moderate/high-intensity exercise and exercising for ≥150-minute per week were protective factors against moderate and severe NPDR, and exercising ≥3 times per week was protective against severe NPDR (Table S4 in the **Online Supplementary Document**).

### Participants in the cohort analysis

The 3669 patients without DR included in the cohort analysis in 2017 (**Figure 1**) had a mean age of 63.10 (SD = 6.56) years and a mean disease duration of 7.48 (SD = 5.32) years. By 2020, 833 (22.7%) patients developed DR (**Table 1**).

**Table 1:** Baseline characteristics of patients with type 2 diabetes in the cohort analysis (n = 3669)

	n (%)
Age in years*	63.1 (6.56)
Duration of diabetes in years*	7.48 (5.32)
BMI, kg/m^2^*	24.57 (2.95)
Hemoglobin A1c, %*	7.24 (1.1)
Arterial pressure, mmHg*	95.25 (4.32)
Exercise frequency, times/week*	4.59 (2.37)
Time per exercise, min*	50.61 (33.01)
Weekly exercise duration, min*	245.22 (228.39)
DR (yes)	833 (22.7)
Sex (female)	2113 (57.59)
Glycaemic control (accordant)	1538 (41.92)
Overweight (yes)	2566 (69.94)
Control of blood pressure (accordant)	1633 (44.51)
PA (active)	607 (16.54)
Exercise intensity (moderate/high)	862 (23.50)
Exercise frequency (≥3 times/week)	3075 (83.81)
Exercise time (≥150 min/week)	2328 (63.45)
Smoke (yes)	453 (12.35)
Drink (yes)	183 (4.99)
Insulin (used)	358 (9.76)

BMI – body mass index, DR – diabetic retinopathy, PA – physical activity

*Values presented as mean (standard deviation).

In the forward Cox regression model, PA was a protective factor against incident DR (hazard ratio (HR) = 0.777; 95% confidence interval (CI) = 0.638–0.947). Other identified risk factors included longer diabetes duration (HR = 1.021; 95% CI = 1.008–1.033), poor glycaemic control (HR = 1.264; 95% CI = 1.096–1.458), insulin use (HR = 1.458; 95% CI = 1.192–1.784), and a smaller PAVR in 2017 (HR = 0.191; 95% CI = 0.070–0.518).

Grouping based on PA levels during 2017–20 revealed that the active group was younger, had poorer blood pressure control, and contained a higher proportion of males, smokers, and drinkers compared to the inactive group (Table S5 in the **Online Supplementary Document**). No significant differences were observed in diabetes duration, glycated haemoglobin levels, prevalence of overweight, insulin use, or retinal vascular parameters between the groups.

### Effect of PA on the incidence of DR

**Figure 2:**
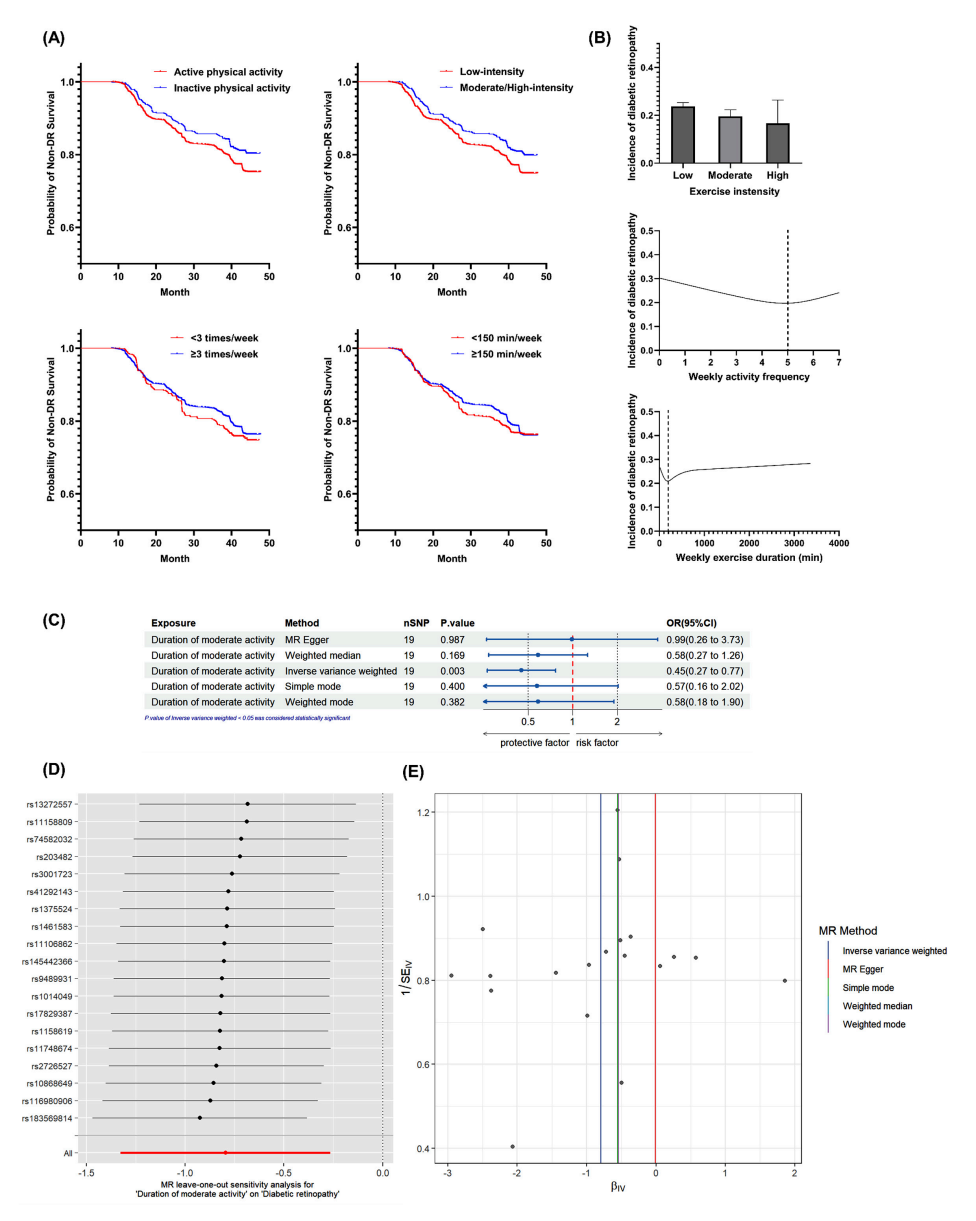
The effect of PA on DR. **Panel A.** Kaplan-Meier curves for DR-free survival by physical activity status and exercise components. **Panel B.** Dose-response relationships between exercise components and DR incidence. **Panel C.** Forest plot of MR analysis for moderate-intensity activity and DR. **Panel D.** Scatter plot of genetic associations for MR analysis. **Panel E.** Leave-one-out sensitivity analysis for MR estimates. DR – diabetic retinopathy, IV – instrumental variable, IVW – inverse variance weighted, MR – Mendelian randomisation, OR – odds ratio, PA – physical activity, SE – standard error, SNP – single-nucleotide polymorphism.

The incidence of DR by 2020 was 18.95% in the active group and 23.45% in the inactive group. Kaplan-Meier analysis indicated a lower cumulative incidence of DR in the active group (**Figure 2**, Panel A; Table S6 in the **Online Supplementary Document**). Moderate/high-intensity exercise reduced cumulative incidence of DR, whereas exercise frequency ≥3 times/week and total exercise time ≥150-minute/week had no significant effect.

PA was a protective factor against the incidence of DR, both in univariate analysis and after multifactorial (age, sex, duration of diabetes, glycaemic control, control of blood pressure, insulin, smoking, drinking, AVR, and PAVR) correction. Moderate/high-intensity exercise showed a protective effect against DR in both univariate and multivariate analyses. However, exercising ≥3 times/week and total exercise duration ≥150-minute/week were not significantly associated with the incidence of DR (**Table 2**). No significant associations were observed for weekly activity frequency, weekly exercise duration, or time per exercise. Moreover, neither PA group (active/inactive) nor exercise components (frequency, duration, or intensity) significantly influenced DR grading in 2020.

**Table 2:** Cox regression analysis of the influence of PA on the incidence of DR

	Unadjusted model		Adjusted model 1*		Adjusted model 2†		Adjusted model 3‡	
	**HR (95% CI)**	***P*-value**	**HR (95% CI)**	***P*-value**	**HR (95% CI)**	***P*-value**	**HR (95% CI)**	***P*-value**
Physically active	0.801 (0.672, 0.956)	0.014	0.808 (0.677, 0.965)	0.019	0.814 (0.681, 0.972)	0.023	0.812 (0.679, 0.971)	0.022
Moderate/high exercise intensity	0.810 (0.694, 0.944)	0.007	0.819 (0.702 0.956)	0.011	0.824 (0.705, 0.963)	0.015	0.823 (0.704, 0.963)	0.015
Exercise frequency ≥3 times/week	0.894 (0.763, 1.049)	0.170	0.890 (0.759, 1.043)	0.151	0.883 (0.752, 1.037)	0.131	0.884 (0.753, 1.038)	0.133
Weekly exercise duration ≥150 min/week	0.944 (0.831, 1.071)	0.369	0.938 (0.826, 1.065)	0.327	0.938 (0.825, 1.066)	0.328	0.938 (0.825, 1.067)	0.332

CI – confidence interval, DR – diabetic retinopathy, HR – hazard ratio, PA– physical activity

*Adjusted for age, sex, and duration of diabetes.

†Adjusted for age, sex, duration of diabetes, overweight, blood pressure control, glycaemic control, insulin use, smoking, and drinking.

‡Adjusted for age, sex, duration of diabetes, overweight, blood pressure control, glycaemic control, insulin, smoking, drinking, arterio-venous ratio, and peripheral arterio-venous ratio.

### Subgroup analysis of exercise components

Among participants performing either low- or moderate/high-intensity exercise, neither frequency (low:* P* = 0.21, moderate/high:* P* = 0.61) nor duration (low:* P* = 0.72, moderate/high:* P* = 0.93) significantly influenced DR risk. Furthermore, among those engaging in moderate/high-intensity exercise, high-intensity exercise showed no significant association with DR risk in either univariate (*P* = 0.50) or multivariate (*P* = 0.48) analyses.

Fitted splines (dose-response curves) revealed a relationship between the incidence of DR and both weekly exercise frequency and duration (**Figure 2**, Panel B). As frequency increased, DR incidence first declined and then rose, reaching its lowest point (DR incidence = 0.197) at 4.67–5.09 sessions/week. Similarly, DR incidence initially decreased with longer weekly exercise duration before increasing, with the minimum (DR incidence = 0.209) occurring at 178.82–193.24-minute/week.

For patients exercising <3 sessions/week, higher exercise frequency significantly reduced DR risk in both univariate (HR = 0.705; 95% CI = 0.532–0.933) and multivariate analyses (HR = 0.698; 95% CI = 0.526–0.926). For those exercising ≥3 sessions/week, moderate/high-intensity exercise lowered DR risk significantly in both univariate (HR = 0.806; 95% CI = 0.670–0.968) and multivariate (HR = 0.812; 95% CI = 0.675–0.977) analyses. Similarly, for patients exercising <5 sessions/week, higher exercise frequency significantly reduced DR risk in both univariate (HR = 0.89; 95% CI = 0.834–0.955) and multivariate (HR = 0.903; 95% CI = 0.843–0.968) analyses. For those exercising ≥5 sessions/week, moderate/high-intensity exercise significantly reduced DR risk in both univariate (HR = 0.688; 95% CI = 0.527–0.898) and multivariate (HR = 0.730; 95% CI = 0.557–0.957) analyses.

For participants exercising <150-minute/week, higher weekly exercise duration exhibited a borderline significant reduction in DR risk after multivariable adjustment (HR = 0.997; 95% CI = 0.993–1.000), although univariate results were not significant (HR = 0.705; 95% CI = 0.532–0.933). For those exercising ≥150-minute/week, moderate/high-intensity exercise significantly decreased DR risk in both univariate (HR = 0.778; 95% CI = 0.633–0.956) and multivariate (HR = 0.789; 95% CI = 0.641–0.972) analyses.

For participants exercising <180-minute/week, higher weekly exercise duration in the univariate (HR = 0.996; 95% CI = 0.994–0.999) and multivariate (HR = 0.997; 95% CI = 0.994–0.999) analysis reduced the DR risk, and higher exercise frequency also reduced it both univariate (HR = 0.897; 95% CI = 0. 834–0.966) and multivariate (HR = 0.910; 95% CI = 0.845–0.980) analysis. Although exercising ≥150-minute/week significantly reduced DR risk in univariate analysis (HR = 0.721; 95% CI = 0.553–0.941), the effect became non-significant after multivariate adjustment (HR = 0.767; 95% CI = 0.587–1.003). For those exercising ≥180-minute/week, moderate/high-intensity exercise significantly decreased DR risk in both univariate (HR = 0.694; 95% CI = 0.558–0.864) and multivariate (HR = 0.719; 95% CI = 0.576–0.898) analyses.

Other exercise components not detailed in the subgroup analyses showed no significant association with the DR.

### Two-sample MR analysis

After SNP selection, 19 SNPs were included (Table S7 in the **Online Supplementary Document**), with an average F-statistic of 23.25, indicating no weak instruments. The IVW method indicated a reduction in DR risk with increased moderate activity duration (odds ratio = 0.45; 95% CI = 0.27–0.77, *P* = 0.003), with directionally consistent but non-significant effects in MR-Egger and weighted median models (**Figure 2**, Panel C). Sensitivity analyses confirmed instrument homogeneity (Cochran’s Q *P* = 0.53), absence of directional pleiotropy (MR-Egger intercept *P* = 0.22), and no outlier SNPs (MR-PRESSO *P* = 0.28). Leave-one-out robustness analysis indicated consistent effects (**Figure 2**, Panel D), supported by funnel plot symmetry (both *P* > 0.05) (**Figure 2**, Panel E).

In reverse MR analysis, we included 649 SNPs. No reverse causality was identified, as all methods (IVW:* P* = 0.89, MR-Egger:* P* = 0.66, and weighted median:* P* = 0.96) yielded non-significant results.

For vigorous-intensity activity analysis, we incorporated 16 SNPs. Similarly, no significant association with DR was observed using IVW (*P* = 0.91), MR-Egger (*P* = 0.92), or weighted median (*P* = 0.24) methods.

### Effect of PA on changes in retinal vessel diameters

In the cross-sectional study, physically active patients had wider arteries (*t* = *−*7.63; *P* = 2.50 × 10^−14^) and veins (*t* = *−*7.32; *P* = 2.49 × 10^−13^); however, there were no significant differences in AVR (*t* = 0.21; *P* = 0.834) or PAVR (*t* = 0.41; *P* = 0.684) between groups.

In the cohort study, physically active patients had a greater increase in the peripheral retinal artery equivalent and PAVR over the three years (Table S5 and Figure S2 in the **Online Supplementary Document**). Linear regression of changes in retinal vessel diameters showed that PA was associated with increased peripheral retinal artery equivalent and PAVR in both univariate and multivariate models, with no significant effect on PRVE, CRAE, CRVE, or AVR (**Table 3**).

**Table 3:** Linear regression analysis of the effect of PA on changes in retinal vascular parameters.

	Univariate regression			Multivariate regression*		
	***β***	**t**	***P*-value**	***Ž***	**t**	***P*-value**
CRAE	−0.269	−0.86	0.389	−0.259	−0.82	0.415
CRVE	−0.272	−0.87	0.385	−0.287	−0.90	0.366
AVR	−0.268	−0.79	0.432	−0.219	−0.63	0.528
PRAE	1.159	2.36	0.018	1.081	2.19	0.029
PRVE	−0.579	−1.64	0.102	−0.121	−0.23	0.815
PAVR	1.118	2.15	0.032	1.050	2.01	0.045

AVR – arterio-venous ratio, CRAE – central retinal artery equivalent, CRVE – central retinal vein equivalent, PA – physical activity, PAVR – peripheral arterio-venous ratio, PRAE – peripheral retinal artery equivalent, PRVE – peripheral retinal vein equivalent

*Adjusted for age, sex, duration of diabetes, overweight, blood pressure control, glycaemic control, insulin use, smoking, and drinking.

## DISCUSSION

We investigated the relationships between PA, retinal vessel diameter, and DR in type 2 diabetes through cohort and cross-sectional studies. While American Diabetes Association PA guidelines may reduce macrovascular risk [1], their effect on microvascular complications, such as DR, remains less clear, warranting further investigation to optimise diabetes management.

### Key findings and comparison with existing literature

Cross-sectionally, patients with mild NPDR were more physically active than those with severe NPDR, aligning with findings that higher activity levels correlate with less severe DR [4]. However, we observed no overall association between PA and DR prevalence, consistent with prior cross-sectional studies [3–6], potentially due to differences in PA definition or confounding factors such as physician advice and socioeconomic status.

Longitudinally, PA demonstrated a protective effect against DR incidence, corroborating prior studies in type 2 diabetes, which found that moderate-to-heavy PA [11] and higher activity levels [12] were associated with reduced DR incidence. This supports the existing American Diabetes Association PA guidelines [1], extending their potential benefits to microvascular eye health.

### Possible mechanisms and explanations

Many factors, such as age, duration of diabetes, glycaemic control, blood pressure, and medication use, influence DR. Exercise, an easily achievable means of health control, can help improve metabolic control of diabetes, especially blood glucose levels [3,4,9]. The metabolic control effect of exercise is beneficial for reducing the progression of DR. However, in our study, poorer blood pressure control was observed in the active group, potentially attributable to a higher proportion of males, smokers, and drinkers.

Although PA did not show a significant metabolic control effect in our study, it still had a protective effect against DR. PA ameliorates the deleterious retinal phenotype [14], in healthy populations [13] and patients with cardiovascular risk [16]. Velayutham and colleagues analysed the retinal vessel diameters outside the central zone (expanded zone) in adolescents with type 1 diabetes and found that the vascular parameters in the expanded zone were independently associated with many DR risk factors [22] and DR progression [23]. Previous cross-sectional studies on type 1 diabetes found that lower PA levels are associated with narrower CRAE [24] and wider CRVE [25]. However, the relationship between PA and retinal vessel diameters in patients with type 2 diabetes remains unclear, as there is a lack of longitudinal studies discussing the relationship. We found that PA significantly widened the peripheral retinal artery equivalent and increased the PAVR longitudinally. PAVR was independently associated with DR prevalence and incidence, suggesting that microvascular changes in smaller peripheral vessels may be an early indicator of DR risk [15,23]. The lack of association with central AVR may relate to the smaller cohort size and shorter follow-up. However, the putative mechanisms underlying PA, DR, and retinal vessel changes warrant validation in extended longitudinal cohorts or controlled animal studies.

### Exercise components

Exercise intensity was a primary factor, with moderate-to-high intensity reducing DR incidence, consistent with other studies [26]. Importantly, subgroup analyses revealed that this benefit was significant only when minimum thresholds of frequency (≥3 sessions/week) and duration (≥150-minute/week) were met. For individuals below these thresholds, increasing frequency or duration was more impactful than increasing intensity. Dose-response curves suggested optimal benefits at approximately ≥5 sessions/week and ≥180-minute/week. Furthermore, among those performing moderate/high-intensity exercise, high-intensity activity provided no significant additional benefit over moderate intensity. Additionally, we employed MR analysis for the first time to validate the relationship between PA and DR, highlighting the importance of moderate-intensity activity in developing exercise prescriptions for DR patients.

### Strengths and limitations

We conducted a large-scale design and longitudinal follow-up study with objective retinal vascular measurements, applying a comprehensive analysis of exercise components combined with MR for causal inference. However, several additional limitations should be acknowledged: First, patient-reported PA data are subject to recall and cognitive biases. Furthermore, PA data did not differentiate between specific exercise types and focussed on prevalent exercises, potentially leading to an underestimation of true activity levels. Caution is needed when interpreting findings on high-intensity exercise, as only 2.6% of participants engaged in this level. Moreover, the findings may not be generalisable to populations with higher engagement in high-intensity exercise and should be applied with caution in diverse settings with varying activity patterns. The three-year observation period may have been insufficient to fully capture DR progression or vascular changes. The specific mechanisms by which PA affects DR and vascular parameters remain to be experimentally or mechanistically validated. Additionally, the exclusion of patients with baseline DR and the lack of annual DR severity assessments (evaluation only in 2020) limited the analysis of disease progression. Unmeasured socioeconomic confounders such as occupation, income, or education may have introduced bias. Lower socioeconomic status is linked to factors that increase DR risk, including poorer nutrition [27] and reduced healthcare access [28]. Meanwhile, higher PA levels may cluster with healthier overall behaviours [28]. Neglecting these confounding factors may lead to an overestimation of the protective effect of exercise on DR. Furthermore, focussing on a Chinese population may limit generalisability. However, the MR analysis utilised European data sets, creating ethnic heterogeneity across study components.

### Implications for practice and future research

The population in Shanghai, China, faces limited access to outdoor recreational spaces within its dense metropolitan environment [29]. Many Chinese patients with diabetes recognise the value of PA but tend to engage predominantly in low-intensity exercise due to barriers like household duties and environmental constraints [30], which likely diminishes its health benefits. Support from family, friends, and the community is crucial for improving self-management [31,32]. Cultural backgrounds significantly influence PA habits, support systems, and barriers [33,34]. Future research should address practical barriers and cultural factors to promote effective PA.

## CONCLUSIONS

In the Chinese population with type 2 diabetes, adhering to the American Diabetes Association PA guidelines significantly reduced DR incidence. The protective effect was influenced by a complex interplay of exercise intensity, frequency, and duration, with benefits optimising at higher thresholds (five sessions and 180-minute/week). Since most patients met frequency and duration targets but only a minority engaged in moderate/high-intensity activity, promoting higher-intensity exercise is essential. PA was associated with beneficial retinal microvascular changes, particularly in peripheral areas. The physiological mechanisms linking PA to retinal health require further investigation in longer-term and experimental studies.

## Additional material


Online Supplementary Document
