# Supplementary material for: The effects of physical activity on diabetic retinopathy in type 2 diabetes using automated vascular analysis: a cohort study
Source: J Glob Health. 2025 Dec 5;15:04319. doi: 10.7189/jogh.15.04319 (PMC12677239; doi:10.7189/jogh.15.04319)
Supplement: Online Supplementary Document [file jogh-15-04319-s001.pdf]

**Supplement to: Xiang Z, Lin S, Xu Y, Lu L, Shi Y, Wang Y, Yang Q, Ling S, Zhou D, Qin X, Cheng M, Zou H, Ma Y. The effects of physical activity on diabetic retinopathy in type 2 diabetes using automated vascular analysis: a cohort study. J Glob Health. 2025;15:04319.**

**Effect of physical activity on diabetic retinopathy and retinal vessel diameters in type 2 diabetes: a cohort study**

Zhaoyu Xiang, MD[a,b]; Senlin Lin, MSc[a]; Yi Xu, PhD[a]; Lina Lu, MPH[a]; Yan Shi, MPH[c,d]; Yuheng Wang, MPH[c]; Qinping Yang, MPH[c]; Saiguang Ling, MSc[e]; Dengji Zhou, MSc[e]; Xinran Qin, BM [a,b]; Minna Cheng, MPH[c*]; Haidong Zou, PhD[a,b *]; Yingyan Ma, PhD[a,b*]

[a] Department of Eye Disease Control and Prevention, Shanghai Eye Disease Prevention and Treatment Center/Shanghai Eye Hospital, Shanghai, China, No. 1440, Hongqiao Road, Shanghai, China

[b] Department of Ophthalmology, Shanghai General Hospital, School of Medicine, Shanghai Jiao Tong University, No. 85/86, Wujin Road, Shanghai, China

[c] Department of Chronic Non-Communicable Diseases and Injury, Shanghai Municipal Centers for Disease Control & Prevention, NO. 1380, West Zhongshan Road, 200336, Shanghai, China

[d] National Clinical Research Center for Aging and Medicine, Huashan Hospital, Fudan University, NO. 12, Middle Wulumuqi Road, 200031, Shanghai, China

[e] Evision Technology (Beijing) Co., Ltd., Beijing 100085, China

[*] **Correspondence to**

Yingyan Ma,

No. 100 Haining Road, Shanghai, 200040, China.

Email: mYy_29@163.com

Haidong Zou

No. 100 Haining Road, Shanghai, 200040, China.

Email: zouhaidong@sjtu.edu.cn

Minna Cheng

No. 1380 West Zhongshan Road, Shanghai, 200336, China

Email: chengminna@scdc.sh.cn

**Table S1. Relationship between physical activity and diabetic retinopathy in previous clinical studies**

| Author | Country | Participant | Number | Physical activity | Follow-up time (years) | Conclusion | The influence of physical activity (HR/OR) |
|---|---|---|---|---|---|---|---|
| Cruickshanks et al. 1995[1] | U.S.A. | Type I | 578 | Questionnaire (leisure-time sports activities in the past week/year, energy expenditure per week) | 6 | No association with development of DR | Vigorous self-rated activity (OR)<br>Development of DR: 1·00 (0·52-1·93); Incidence of PDR: 0·68 (0·27-1·73) |
| Ahmed et al. 2011[2] | Bangladesh | Type II | 977 | Physical activity at work | 15 | a protective factor for the incidence of DR after 5 years | Moderate to heavy (IRR): 0·21 (0·13–0·33) |
| Makura et al. 2013[3] | U.S.A. and Canada | Type I | 1,441 | Questionnaire (LTPA of different intensities in the past 7 days, energy expenditure per week) | 6·5 (3-9) | No association with development of DR | Energy expenditure per week (HR)<br>450-1500METs: 0·93 (0·41-2·09);<br>>1500METs: 1·13 (0·73-1·77) |
| Kuwata et al. 2017[4] | Japan | Type II | 1,814 | IPAQ (energy expenditure per week) | 2 | Higher PA level was independently associated with a lower incidence of DR | PA categories (HR)<br>Second: 0·87 (0·53-1·40); Third: 0·83 (0·52-1·31); Fourth: 0·58 (0·35-0·94); Fifth: 0·63 (0·42-0·94) |
| Tikkanen-Dolenc et al. 2020[5] | Finland | Type I | 1,612 | KIHD questionnaire (LTPA) | 10·7 ± 4·6 | Frequent LTPA was associated with a lower incidence of severe diabetic retinopathy* | <2 sessions/week (HR)<br>Univariate: 1·40 (1·03–1·89); Multivariate: 1·18 (0·85–1·62) |
| Yan et al. 2021[6] | Australia | 45-65 years old | 9,018 | AAS questionnaire (PA categories based on the MET-adjusted session) | 10 | Higher PA level was independently associated with a lower risk of DR progression* | PA categories (HR)<br>Medium: 0·78 (0·61-0·98); High: 0·61 (0·36-0·84) |

**Table S1. Relationship between physical activity and diabetic retinopathy in previous clinical studies**

| | | | | | | | |
|---|---|---|---|---|---|---|---|
| LaPorte et al. 1986[7] | U.S.A. | Type I | 696 | Questionnaire from the Harvard Alumni Study (PA during the past week, historical leisure activity) | Cross-sectional | No association with prevalence of severe retinopathy | N/A |
| Kriska et al. 1991[8] | U.S.A. | Type I | 628 | Questionnaire from the Harvard Alumni Study (PA during the past week, historical leisure activity) | Cross-sectional | No association with prevalence of PDR | N/A |
| Cruickshanks et al. 1992[9] | U.S.A. | Type I, <40 years old | 818 | Questionnaire (LTPA in the past week/year, energy expenditure per week) | Cross-sectional | Higher levels of PA may be associated with a reduced risk of having PDR in women, not in men | Women (diagnosed<14 years) reported a historical leisure activity (OR): 0·46 (0·23-0·93)<br>women vigorous self-rated activity (OR): 0·34 (0·13-0·87) |
| Wadén et al. 2008[10] | Finland | Type I | 1,945 | KIHD questionnaire (LTPA) | Cross-sectional | Low-intensity LTPA, but not total or low-frequency LTPA, was independently associated with PDR | Low-intensity LTPA (OR): 1·49 (1·15-1·93) |
| Tikellis et al. 2010[11] | U.S.A. | 45-64 years old | 12,363 | Modified Baecke Physical Activity questionnaire (PA performed during leisure, sport and work times) | Cross-sectional | Higher work-related PA level was associated with a lower prevalence of DR | Work-related PA above the median (OR): 0·66 (0·51-0·85) |
| Kim et al. 2011[12] | Korea | Type II,<br>>40 years old | 1,298 | Questionnaire (regular exercise) | Cross-sectional | No association with prevalence of DR | N/A |
| Li et al. 2013[13] | China | Type II | 10,100 | Questionnaire (regular exercise) | Cross-sectional | No regular exercise was a risk factor for DR | No regular exercise (OR): 1·42 (1·04-1·94) |

**Table S1. Relationship between physical activity and diabetic retinopathy in previous clinical studies**

| | | | | | | | |
|---|---|---|---|---|---|---|---|
| Yang et al. 2013[14] | Korea | ≥19 years old | 998 | Nationally representative survey (regular exercise: ≥5 sessions/week) | Cross-sectional | No association with prevalence of DR | N/A |
| Bener et al. 2014[15] | Qatar | >20 years old | 1,633 | Questionnaire (regular exercise) | Cross-sectional | Regular exercise was a risk factor for DR | Regular exercise (OR): 1·91 (1·30–2·82) |
| Wang et al. 2014[16] | China | BMI>25, Type II | 2,699 | Medical interview (regular exercise: ≥30min of moderate or vigorous activity per day at least 3 days per week) | Cross-sectional | No association with prevalence of DR | Regular exercise (OR): 1·03 (0·85–1·24) |
| Bohn et al. 2015[17] | Germany and Austria | Type I | 18,028 | Self-reported PA (the average time engaged in specific activities during the past year) | Cross-sectional | Low frequency of activity increases the prevalence of DR | N/A |
| Li et al. 2015[18] | China | Type II | 517 | Self-reported frequency of PA (PA0, inactive; PA1, 1-2 sessions/week; PA2, >2 sessions/week) | Cross-sectional | Moderate-intensity PA was a protective factor for DR | Moderate-intensity PA (OR): 0·57 (0·37–0·89) |
| Loprinzi 2016[19] | U.S.A. | >20 years old | 282 | Accelerometer (average min/day; sedentary behavior: activity counts <100/min) | Cross-sectional | Increase in sedentary time was a risk factor for mild or worse DR, and total PA time was not associated with prevalence of DR | Sedentary time (OR):1·16 (1·00-1·35); total PA time (OR): 1·00 (0·99-1·01) |
| Yan and Ma 2016[20] | China | Type II | 1,100 | Medical interview (routine physical exercise, exercise type) | Cross-sectional | No association with prevalence of DR | Walking (OR): 0·71 (0·13-3·87) |

## Table S1. Relationship between physical activity and diabetic retinopathy in previous clinical studies

| | | | | | | | |
|---|---|---|---|---|---|---|---|
| Praidou et al. 2017[21] | U.K. | 240 DR & 80 Control Type II | 320 | IPAQ (energy expenditure per week) | Cross-sectional | Increased PA is associated with less severe levels of DR | PA (OR): 0·73 (0·66–0·80) |
| Dharmastuti et al. 2018[22] | Indonesia | Type II | 1,116 | GPAQ (total energy expenditure for 24 hours) | Cross-sectional | Sedentary activity ≥ 3h/day was strongly associated with prevalence of DR and Vision-Threatening DR** | Sedentary activity ≥ 3h/day (OR) DR: 1·66 (1·17-2·35); Vision-Threatening DR: 1·74 (1·16-2·62) |
| Wang et al. 2019[23] | China | >50 years old | 3,031 | Standardized questionnaire (energy expenditure per week) | Cross-sectional | Higher PA was associated with a lower prevalence of DR | PA (non-standardizedβ): -15·7 (-27·6, -3·90) |
| Martin et al. 2021[24] | Argentina | Type II | 495 | IPAQ (energy expenditure per week) | Cross-sectional | Low levels of PA were associated with prevalence of DR | N/A |

Hazard ratio (HR), odds ratio (OR), physical activity (PA), leisure-time PA (LTPA), body mass index (BMI), not applicable (N/A), diabetic retinopathy (DR), proliferative diabetic retinopathy (PDR), metabolic equivalent of task (MET), International Physical Activity Questionnaire (IPAQ), Kuopio Ischaemic Heart Disease Risk Factor Study (KIHD), Active Australia Survey (AAS), Global Physical Activity Questionnaire (GPAQ); *The initiation of laser treatment due to severe non-proliferative DR, proliferative DR, or diabetic maculopathy; ** Vision-threatening DR included severe non-proliferative DR, proliferative DR, and clinically significant macular oedema

## References


1. Cruickshanks KJ, Moss SE, Klein R, Klein BE. Physical activity and the risk of progression of retinopathy or the development of proliferative retinopathy. *Ophthalmology*. Aug 1995;102(8):1177-82. doi:10.1016/s0161-6420(95)30893-7
2. Ahmed KR, Karim MN, Bukht MS, et al. Risk factors of diabetic retinopathy in Bangladeshi type 2 diabetic patients. *Diabetes Metab Syndr*. Oct-Dec 2011;5(4):196-200. doi:10.1016/j.dsx.2012.02.020
3. Makura CB, Nirantharakumar K, Girling AJ, Saravanan P, Narendran P. Effects of physical activity on the development and progression of microvascular complications in type 1 diabetes: retrospective analysis of the DCCT study. *BMC Endocr Disord*. Oct 2 2013;13:37. doi:10.1186/1472-6823-13-37
4. Kuwata H, Okamura S, Hayashino Y, Tsujii S, Ishii H. Higher levels of physical activity are independently associated with a lower incidence of diabetic retinopathy in Japanese patients with type 2 diabetes: A prospective cohort study, Diabetes Distress and Care Registry at Tenri (DDCRT15). *PLoS One*. 2017;12(3):e0172890. doi:10.1371/journal.pone.0172890
5. Tikkanen-Dolenc H, Wadén J, Forsblom C, et al. Frequent physical activity is associated with reduced risk of severe diabetic retinopathy in type 1 diabetes. *Acta Diabetol*. May 2020;57(5):527-534. doi:10.1007/s00592-019-01454-y
6. Yan X, Han X, Wu C, Shang X, Zhang L, He M. Effect of physical activity on reducing the risk of diabetic retinopathy progression: 10-year prospective findings from the 45 and Up Study. *PLoS One*. 2021;16(1):e0239214. doi:10.1371/journal.pone.0239214
7. LaPorte RE, Dorman JS, Tajima N, et al. Pittsburgh Insulin-Dependent Diabetes Mellitus Morbidity and Mortality Study: physical activity and diabetic complications. *Pediatrics*. Dec 1986;78(6):1027-33.
8. Kriska AM, LaPorte RE, Patrick SL, Kuller LH, Orchard TJ. The association of physical activity and diabetic complications in individuals with insulin-dependent diabetes mellitus: the Epidemiology of Diabetes Complications Study--VII. *J Clin Epidemiol*. 1991;44(11):1207-14. doi:10.1016/0895-4356(91)90153-z
9. Cruickshanks KJ, Moss SE, Klein R, Klein BE. Physical activity and proliferative retinopathy in people diagnosed with diabetes before age 30 yr. *Diabetes Care*. Oct 1992;15(10):1267-72. doi:10.2337/diacare.15.10.1267
10. Wadén J, Forsblom C, Thorn LM, et al. Physical activity and diabetes complications in patients with type 1 diabetes: the Finnish Diabetic Nephropathy (FinnDiane) Study. *Diabetes Care*. Feb 2008;31(2):230-2. doi:10.2337/dc07-1238
11. Tikellis G, Anuradha S, Klein R, Wong TY. Association between physical activity and retinal microvascular signs: the Atherosclerosis Risk in Communities (ARIC) Study. *Microcirculation*. Jul 2010;17(5):381-93. doi:10.1111/j.1549-8719.2010.00033.x
12. Kim JH, Kwon HS, Park YM, et al. Prevalence and associated factors of diabetic retinopathy in rural Korea: the Chungju metabolic disease cohort study. *J Korean Med Sci*. Aug 2011;26(8):1068-73. doi:10.3346/jkms.2011.26.8.1068
13. Li N, Yang XF, Deng Y, et al. [Diabetes self-management and its association with diabetic retinopathy in patients with type 2 diabetes]. *Zhonghua Yan Ke Za Zhi*. Jun 2013;49(6):500-6.
14. Yang JY, Kim NK, Lee YJ, et al. Prevalence and factors associated with diabetic retinopathy in a Korean adult population: the 2008-2009 Korea National Health and Nutrition Examination Survey. *Diabetes Res Clin Pract*. Dec 2013;102(3):218-24. doi:10.1016/j.diabres.2013.10.016
15. Bener A, Al-Laftah F, Al-Hamaq AO, Daghash M, Abdullatef WK. A study of diabetes complications in an endogamous population: an emerging public health burden. *Diabetes Metab Syndr*. Apr-Jun 2014;8(2):108-14. doi:10.1016/j.dsx.2014.04.005
16. Wang J, Chen H, Zhang H, et al. The performance of a diabetic retinopathy risk score for screening for diabetic retinopathy in Chinese overweight/obese patients with type 2 diabetes mellitus. *Ann Med*. Sep 2014;46(6):417-23. doi:10.3109/07853890.2013.878977
17. Bohn B, Herbst A, Pfeifer M, et al. Impact of Physical Activity on Glycemic Control and Prevalence of Cardiovascular Risk Factors in Adults With Type 1 Diabetes: A Cross-sectional Multicenter Study of 18,028 Patients. *Diabetes Care*. Aug 2015;38(8):1536-43. doi:10.2337/dc15-0030
18. Li Y, Wu QH, Jiao ML, et al. Gene-environment interaction between adiponectin gene polymorphisms and environmental factors on the risk of diabetic retinopathy. *J Diabetes Investig*. Jan 2015;6(1):56-66. doi:10.1111/jdi.12249
19. Loprinzi PD. Association of Accelerometer-Assessed Sedentary Behavior With Diabetic

Retinopathy in the United States. *JAMA Ophthalmol*. Oct 1 2016;134(10):1197-1198. doi:10.1001/jamaophthalmol.2016.2400
20. Yan ZP, Ma JX. Risk factors for diabetic retinopathy in northern Chinese patients with type 2 diabetes mellitus. *Int J Ophthalmol*. 2016;9(8):1194-9. doi:10.18240/ijo.2016.08.17
21. Praidou A, Harris M, Niakas D, Labiris G. Physical activity and its correlation to diabetic retinopathy. *J Diabetes Complications*. Feb 2017;31(2):456-461. doi:10.1016/j.jdiacomp.2016.06.027
22. Dharmastuti DP, Agni AN, Widyaputri F, et al. Associations of Physical Activity and Sedentary Behaviour with Vision-Threatening Diabetic Retinopathy in Indonesian Population with Type 2 Diabetes Mellitus: Jogjakarta Eye Diabetic Study in the Community (JOGED.COM). *Ophthalmic Epidemiol*. Apr 2018;25(2):113-119. doi:10.1080/09286586.2017.1367410
23. Wang YX, Wei WB, Xu L, Jonas JB. Physical activity and eye diseases. The Beijing Eye Study. *Acta Ophthalmol*. May 2019;97(3):325-331. doi:10.1111/aos.13962
24. Martin CG, Pomares ML, Muratore CM, et al. Level of physical activity and barriers to exercise in adults with type 2 diabetes. *AIMS Public Health*. 2021;8(2):229-239. doi:10.3934/publichealth.2021018

**Checklist S1. STROBE Statement—checklist of items that should be included in reports of observational studies**

| | Item No | Recommendation |
|---|---|---|
| **Title and abstract** | 1 | (*a*) Indicate the study's design with a commonly used term in the title or the abstract **Yes, page 1 and 3** |
| | | (*b*) Provide in the abstract an informative and balanced summary of what was done and what was found **Yes, page 3** |
| **Introduction** | | |
| Background/rationale | 2 | Explain the scientific background and rationale for the investigation being reported **Yes, page 5** |
| Objectives | 3 | State specific objectives, including any prespecified hypotheses **Yes, page 5 (line 100-105) and page 6 (line 106-111)** |
| **Methods** | | |
| Study design | 4 | Present key elements of study design early in the paper **Yes, page 6 (line 114-120)** |
| Setting | 5 | Describe the setting, locations, and relevant dates, including periods of recruitment, exposure, follow-up, and data collection **Yes, figure 1 and page 6 (line 114-120)** |
| Participants | 6 | (*a*) *Cohort study*—Give the eligibility criteria, and the sources and methods of selection of participants. Describe methods of follow-up *Case-control study*—Give the eligibility criteria, and the sources and methods of case ascertainment and control selection. Give the rationale for the choice of cases and controls *Cross-sectional study*—Give the eligibility criteria, and the sources and methods of selection of participants **Yes, figure 1 and Supplementary file (Methods and Statistical analysis, page 3, line 34-45)** |
| | | (*b*) *Cohort study*—For matched studies, give matching criteria and number of exposed and unexposed *Case-control study*—For matched studies, give matching criteria and the number of controls per case **Not applicable.** |
| Variables | 7 | Clearly define all outcomes, exposures, predictors, potential confounders, and effect modifiers. Give diagnostic criteria, if applicable **Yes, table 1 (page 7) and Supplementary file (Methods and Statistical analysis, page 3, line 48-53; page 4, line 66-69; page5-6, line 90-100)** |
| Data sources/ measurement | 8* | For each variable of interest, give sources of data and details of methods of assessment (measurement). Describe comparability of assessment methods if there is more than one group **Yes, Supplementary file (Methods and Statistical analysis, page 3-6, line 47-100)** |
| Bias | 9 | Describe any efforts to address potential sources of bias **Yes, Supplementary file (Methods and Statistical analysis, page 6, line 108-118)** |
| Study size | 10 | Explain how the study size was arrived at **Yes, figure 1 and Supplementary file (Methods and Statistical analysis, page 3,** |

| | | |
|---|---|---|
| | | **line 34-45)** |
| Quantitative variables | 11 | Explain how quantitative variables were handled in the analyses. If applicable, describe which groupings were chosen and why<br>**Yes, Supplementary file (Methods and Statistical analysis, page 6, line 103-118)** |
| Statistical methods | 12 | (*a*) Describe all statistical methods, including those used to control for confounding<br>**Yes, Supplementary file (Methods and Statistical analysis, page 6, line 108-118)** |
| | | (*b*) Describe any methods used to examine subgroups and interactions<br>**Yes, Supplementary file (Methods and Statistical analysis, page 6, line 108-118)** |
| | | (*c*) Explain how missing data were addressed<br>**Yes, Supplementary file (Methods and Statistical analysis, page 3, line 41-43)** |
| | | (*d*) *Cohort study*—If applicable, explain how loss to follow-up was addressed<br>*Case-control study*—If applicable, explain how matching of cases and controls was addressed<br>*Cross-sectional study*—If applicable, describe analytical methods taking account of sampling strategy<br>**Yes, Supplementary file (Methods and Statistical analysis, page 3, line 41-43)** |
| | | (*e*) Describe any sensitivity analyses<br>**Not applicable.** |

**Results**

| | | |
|---|---|---|
| Participants | 13* | (a) Report numbers of individuals at each stage of study—eg numbers potentially eligible, examined for eligibility, confirmed eligible, included in the study, completing follow-up, and analysed<br>**Yes, figure 1 and Supplementary file (Methods and Statistical analysis, page 3, line 34-45)** |
| | | (b) Give reasons for non-participation at each stage<br>**Yes, figure 1 and Supplementary file (Methods and Statistical analysis, page 3, line 34-45)** |
| | | (c) Consider use of a flow diagram<br>**Yes, figure 1** |
| Descriptive data | 14* | (a) Give characteristics of study participants (eg demographic, clinical, social) and information on exposures and potential confounders<br>**Yes, table 1 (page 7-8)** |
| | | (b) Indicate number of participants with missing data for each variable of interest<br>**Yes, figure 1 and Supplementary file (Methods and Statistical analysis, page 3, line 34-45)** |
| | | (c) *Cohort study*—Summarise follow-up time (eg, average and total amount)<br>**Yes, figure 1 and Supplementary file (Methods and Statistical analysis, page 3, line 34-45)** |
| Outcome data | 15* | *Cohort study*—Report numbers of outcome events or summary measures over time<br>**Yes, page 8-9 (159-166)** |
| | | *Case-control study*—Report numbers in each exposure category, or summary measures of exposure |
| | | *Cross-sectional study*—Report numbers of outcome events or summary measures<br>**Yes, page 6-8 (126-139)** |
| Main results | 16 | (*a*) Give unadjusted estimates and, if applicable, confounder-adjusted estimates and their precision (eg, 95% confidence interval). Make clear which confounders were adjusted for and |

| | | |
|---|---|---|
| | | why they were included<br>**Yes, page 9-12 (168-206)** |
| | | (*b*) Report category boundaries when continuous variables were categorized<br>**Yes, supplementary file (Methods and Statistical analysis, page 3, line 48-53; page 4, line 66-69)** |
| | | (*c*) If relevant, consider translating estimates of relative risk into absolute risk for a meaningful time period |
| Other analyses | 17 | Report other analyses done—eg analyses of subgroups and interactions, and sensitivity analyses<br>**Yes, supplementary file (table S3 and S4)** |
| **Discussion** | | |
| Key results | 18 | Summarise key results with reference to study objectives<br>**Yes, page 12-13 (line 213-225, 241-248)** |
| Limitations | 19 | Discuss limitations of the study, taking into account sources of potential bias or imprecision. Discuss both direction and magnitude of any potential bias<br>**Yes, page 14 (line 260-265)** |
| Interpretation | 20 | Give a cautious overall interpretation of results considering objectives, limitations, multiplicity of analyses, results from similar studies, and other relevant evidence<br>**Yes, page 14 (line 266-272)** |
| Generalisability | 21 | Discuss the generalisability (external validity) of the study results<br>**Yes, page 14 (line 260-265)** |
| **Other information** | | |
| Funding | 22 | Give the source of funding and the role of the funders for the present study and, if applicable, for the original study on which the present article is based<br>**Yes, page 17 (line 348-355)** |

*Give information separately for cases and controls in case-control studies and, if applicable, for exposed and unexposed groups in cohort and cross-sectional studies.

**Note:** An Explanation and Elaboration article discusses each checklist item and gives methodological background and published examples of transparent reporting. The STROBE checklist is best used in conjunction with this article (freely available on the Web sites of PLoS Medicine at http://www.plosmedicine.org/, Annals of Internal Medicine at http://www.annals.org/, and Epidemiology at http://www.epidem.com/). Information on the STROBE Initiative is available at www.strobe-statement.org.

## **Text S1** MATERIALS AND METHODS

### Study participants

Patients with type 2 diabetes were selected from the SCODE cohort (2017–2020). The study adhered to the principles of the Declaration of Helsinki and was approved by the Ethics Committee of Shanghai First People's Hospital (2013KY023; clinicaltrials.gov: NCT03665090). Participants provided informed consent. In 2017, fundus photographs, PA data, and anthropometric data were collected. However, 4,783 patients with ocular comorbidities other than DR were excluded, resulting in 42,992 patients for cross-sectional analysis. The SCODE study's inclusion and exclusion criteria have been previously reported (17). From 2017–2020, 5,056 patients without DR were followed-up annually, with fundus photographs taken in 2020. Of 3,835 patients with 3-year consistent PA data, 166 lacking anthropometric data were excluded, leaving 3,669 patients in the cohort analysis (Figure 1). Anthropometric and PA data in 2017 were regarded as the baseline values, whereas those from 2017 to 2020 were expressed as 3-year averages of the examination results.

### Anthropometric measurements

The patients' anthropometric measurements (weight, height, body mass index [BMI], blood pressure, fasting glucose, and HbA1c) were obtained using standard procedures. The details of the examinations in the SCODE cohort have been described previously (1). Overweight was defined based on a BMI cut-off ≥23 kg/m$^2$ (2). The cut-off value considered for good blood pressure control was <130/80 mmHg (3). Good glycaemic control was defined as a glucose level of 4.4–7.2 mmol/L for fasting and <7% for HbA1c (4).

**Physical activity**

The physician interviewed the patients to document their weekly exercise habits. The intensity, frequency, and time per session of the longest LTPA performed by the patient per week were recorded. If several episodes of exercises of the same intensity were performed separately, the time and frequency of each exercise were accumulated. Total exercise duration = exercise frequency × time per session.

Low-intensity exercise was considered to involve no self-reported subjective shortness of breath and no sweating; moderate-intensity exercise was considered to require a moderate amount of effort, noticeably accelerating the heart rate; and high-intensity exercise was considered to require a large amount of effort, causing rapid breathing and a substantial increase in heart rate during physical activities (5).

According to the American Diabetes Association Guidelines (6), patients who engage in exercise ≥3 times/week, moderate-intensity exercise ≥150 min/week, or high-intensity exercise ≥75 min/week were considered to be physically active. Only participants with the same PA status in each of the 3 years were included in the cohort study.

**Fundus photographs and retinal vessel diameters**

Retinal photographs were obtained using standardised methods. For each participant, after 5 min of dark adaptation, the optic disc and macula of both eyes were photographed in a darkened room using a 45° 3.78-megapixel digital non-mydriatic camera (Topcon NW400, Topcon, Tokyo, Japan).

The diagnosis of DR was made by physicians based on fundus photography of the macula according to the International Clinical Classification of DR proposed at the 2002 International Ophthalmology Conference (7). Optic disc-centred photos of the right eye were used for analyses; if they were lacking, photos of the left eye were used instead.

This automated method provided precise measurements with a deviation of <5%, compared with manual measurements (8). First, the target area was obtained by region-of-interest extraction, and the difference between images was reduced by methods including denoising, normalisation, and image enhancement. (8). The pre-processed fundus image was inputted into the deep learning network ResNet101-UNet for vascular segmentation. Then, the deep learning network and the edge extraction algorithm based on the visual attention mechanism were combined to locate and segment the optic disk (8). The diameter of the optic disk was defined based on the diameter of the smallest external circle in the optic disc segmentation area, and the blood vessel diameter was calculated automatically using the optical disk diameter as a reference (9).

The modified Parr-Hubbard formula (9) was used for the iterative calculation of the CRAE (intraclass correlation coefficient [ICC]: 0.860 [0.787–0.909]) and CRVE (ICC: 0.805 [0.703–0.872]), which were subsequently divided to calculate the AVR (ICC: 0.807 [0.705–0.874]) (9). The CRAE and CRVE represent the average calibres of the six largest retinal arteries and veins within a region of 0.5–1.0 optic disc diameter from the margin of the optic disc, respectively. The peripheral retinal artery equivalent (PRAE; ICC: 0.915 [0.871–0.945]), vein equivalent (PRVE; ICC: 0.789 [0.678–0.862]), and arteriole-to-venule ratio (PAVR; ICC: 0.801 [0.696–0.870]) were similarly calculated for a region of 1.5–2.0 optic disc diameters

from the optic disc (Figure S1). Changes in retinal vessel diameters (ΔCRAE, CRVE, AVR, PRAE, PRVE, and PAVR) were calculated as the ratio of the difference in retinal vessel diameters (2020–2017) compared with the baseline (2017).

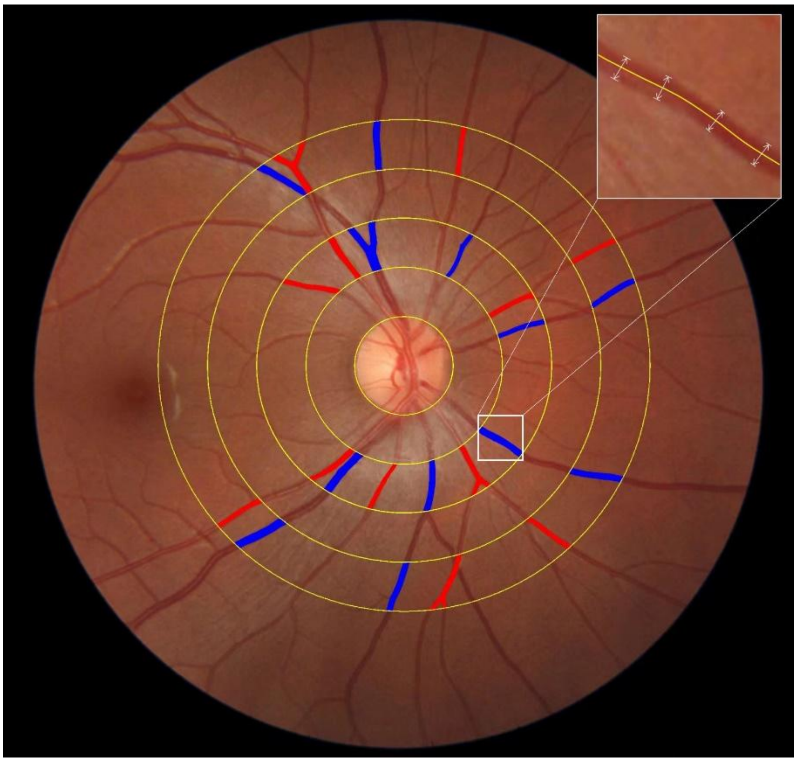

**Figure S1. Measurement of retinal vascular parameters**

The yellow circles outline the detection area of the retinal vessels, with each circle spaced 0.5 optic disc diameter apart. Retinal arteries and veins are marked in red and blue, respectively. The specific vessel detection zone is shown as an enlarged square box.

The segmented blood vessels were treated with morphological erosion to obtain the centreline of the vessels. An orthogonal straight-line tangent at a point on the centreline intersected two points at the edge of the blood vessel, and the distance between the two points was the diameter of the blood vessel corresponding to that point on the centreline.

**Statistical analysis**

Statistical analysis used IBM SPSS Statistics for Windows, version 22 (IBM Corp., Armonk, NY, USA), with significance set at $p < 0.05$. Continuous variables were presented as 'mean±standard deviation'; binary variables as 'percentages in classes 1 and 2'. Due to

significant correlation between left and right eye data ($p < 0.05$), only right-eye data were analysed.

Independent t-tests and chi-square tests compared continuous and categorical variables between groups, respectively. One-way ANOVA assessed between-group differences, with Bonferroni test for post-hoc comparisons. Multiple linear regression analysed factors influencing retinal vessel diameters. A logistic regression model was used to analyse the contributions of different influencing factors to the prevalence of DR, non-proliferative DR (NPDR), and proliferative DR (PDR). The Kaplan–Meier test was used to analyse the contribution of PA to the incidence of DR. A Cox regression model was used to analyse the contribution of different influencing factors to the incidence of DR. The inclusion criterion for independent variables in the regression equation was $p \leq 0.05$, while the independent variable exclusion criterion was $p \geq 0.10$. When determining sex contribution, males and females were assigned 1 and 0, respectively.

## Reference

1. Peng J, Zou H, Wang W, Fu J, Shen B, Bai X, et al. Implementation and first-year screening results of an ocular telehealth system for diabetic retinopathy in China. BMC Health Serv Res. 2011;11:250.
2. Draznin B, Aroda VR, Bakris G, Benson G, Brown FM, Freeman R, et al. 8. Obesity and Weight Management for the Prevention and Treatment of Type 2 Diabetes: Standards of Medical Care in Diabetes-2022. Diabetes Care. 2022;45(Suppl 1):S113-s24.
3. 10. Cardiovascular Disease and Risk Management: Standards of Medical Care in Diabetes-2022. Diabetes Care. 2022;45(Suppl 1):S144-s74.
4. American Diabetes Association Professional Practice C. 6. Glycemic Targets: Standards of Medical Care in Diabetes-2022. Diabetes Care. 2022;45(Suppl 1):S83-S96.
5. Tikkanen-Dolenc H, Wadén J, Forsblom C, Harjutsalo V, Thorn LM, Saraheimo M, et al. Frequent physical activity is associated with reduced risk of severe diabetic retinopathy in type 1 diabetes. Acta Diabetol. 2020;57(5):527-34.
6. Draznin B, Aroda VR, Bakris G, Benson G, Brown FM, Freeman R, et al. 5. Facilitating Behavior Change and Well-being to Improve Health Outcomes: Standards of Medical Care in Diabetes-2022. Diabetes Care. 2022;45(Suppl 1):S60-s82.
7. Wilkinson CP, Ferris FL, 3rd, Klein RE, Lee PP, Agardh CD, Davis M, et al. Proposed international clinical diabetic retinopathy and diabetic macular edema disease severity scales. Ophthalmology. 2003;110(9):1677-82.
8. Long T, Xu Y, Zou H, Lu L, Yuan T, Dong Z, et al. A Generic Pixel Pitch Calibration Method for Fundus Camera via Automated ROI Extraction. Sensors (Basel). 2022;22(21).
9. Tirsi A, Bruehl H, Sweat V, Tsui W, Reddy S, Javier E, et al. Retinal vessel abnormalities are associated with elevated fasting insulin levels and cerebral atrophy in nondiabetic individuals. Ophthalmology. 2009;116(6):1175-81.

**Table S2. <u>Patients with type 2 diabetes</u> in the SCODE cohort in 2017 (n = 42,992)**

| | **Mean±SD / group (N, %)** |
|---|---|
| Age (year) | 64.42 ± 6.87 |
| Duration of diabetes (year) | 8.25 ± 5.49 |
| Body mass index (kg/m2) | 24.51 ± 2.96 |
| Fasting glucose (mmol/L) | 6.88 ± 1.28 |
| Mean arterial pressure (mmHg) | 95.37 ± 5.9 |
| Exercise frequency (times/week) | 4.46 ± 1.83 |
| Time per exercise (min) | 42.48 ± 20.5 |
| Weekly exercise duration (min) | 188.93 ± 114.35 |
| Diabetic retinopathy (Yes) | 8680, 20.19% |
| Proliferative retinopathy (Yes) | 127, 0.3% |
| Sex (Female) | 23743, 55.23% |
| Control of glucose (Accordant) | 13131, 30.54% |
| Overweight (Yes) | 9578, 22.28% |
| Control of blood pressure (Accordant) | 11833, 27.52% |
| Physical activity (Active) | 8241, 19.17% |
| Exercise intensity (Moderate/High) | 11955, 27.53% |
| Exercise frequency (≥3 times/week) | 37988, 88.36% |
| Exercise time (≥150 min/week) | 26087, 60.68% |
| Smoke (Yes) | 3271, 7.61% |
| Drink (Yes) | 1906, 4.43% |
| Insulin (Used) | 7656, 17.81% |

## Table S3. Factors influencing the prevalence of diabetic retinopathy

| | DR | | Moderate NPDR† | | Severe NPDR‡ | | PDR | |
|---|---|---|---|---|---|---|---|---|
| | **OR (95% CI)** | **p-value** | **OR (95% CI)** | **p-value** | **OR (95% CI)** | **p-value** | **OR (95% CI)** | **p-value** |
| Age | 0.99 (0.987, 0.994) | <0.001* | 1.021 (1.013, 1.028) | <0.001* | 0.986 (0.974, 0.997) | 0.014* | 1.04 (1.013, 1.068) | 0.004* |
| Sex | 0.935 (0.891, 0.982) | 0.007* | N/A | N/A | N/A | N/A | N/A | N/A |
| Duration of diabetes | 1.057 (1.052, 1.061) | <0.001* | 1.017 (1.008, 1.026) | <0.001* | 1.027 (1.014, 1.041) | <0.001* | N/A | N/A |
| Control of glucose | 1.114 (1.053, 1.179) | <0.001* | N/A | N/A | 1.198 (1.008, 1.424) | 0.041* | N/A | N/A |
| Control of blood pressure | 1.156 (1.094, 1.222) | <0.001* | N/A | N/A | 1.227 (1.017, 1.482) | 0.033* | N/A | N/A |
| Insulin | 1.702 (1.606, 1.805) | <0.001* | 1.38 (1.227, 1.551) | <0.001* | 1.358 (1.148, 1.606) | <0.001* | N/A | N/A |
| AVR | 0.118 (0.082, 0.169) | <0.001* | 0.161 (0.08, 0.326) | <0.001* | 0.057 (0.018, 0.183) | <0.001* | 0.01 (0.001, 0.145) | 0.001* |
| PAVR | 0.217 (0.158, 0.3) | <0.001* | 0.362 (0.194, 0.675) | 0.001* | 0.328 (0.117, 0.918) | 0.034* | 0.045 (0.004, 0.525) | 0.013* |
| Active physical activity | N/A | N/A | 0.759 (0.675, 0.852) | <0.001* | 0.627 (0.499, 0.789) | <0.001* | N/A | N/A |
| Smoke | N/A | N/A | 1.489 (1.251, 1.771) | <0.001* | N/A | N/A | N/A | N/A |

Diabetic retinopathy (DR), non-proliferative diabetic retinopathy (NPDR), proliferative diabetic retinopathy (PDR), odds ratio (OR), confidence interval (CI);

A stepwise method was used to construct a binary logistic regression equation to analyze the risk factors for DR, different degrees of NPDR, and PDR; the criterion for the inclusion of independent variables in the equation was $p \leq 0.05$, and the independent variable exclusion criterion of the equation was $p \geq 0.10$.; †compared with mild NPDR patients; ‡compared with moderate NPDR patients; * $p<0.05$, a significantly influencing factor in the regression equation

## Table S4. Effect of exercise elements on the severity of non-proliferative diabetic retinopathy

| | Moderate NPDR unadjusted[a] | | Moderate NPDR adjusted[a] | | Severe NPDR unadjusted[b] | | Severe NPDR adjusted[b] | |
|---|---|---|---|---|---|---|---|---|
| | OR (95% CI) | P-value | OR (95% CI) | P-value | OR (95% CI) | P-value | OR (95% CI) | P-value |
| Active physical activity | 0.711 (0.634, 0.796) | <0.001* | 0.759 (0.675, 0.852) | <0.001* | 0.448 (0.355, 0.566) | <0.001* | 0.485 (0.383, 0.615) | <0.001* |
| Moderate/high exercise intensity | 0.755 (0.681, 0.838) | <0.001* | 0.811 (0.73, 0.901) | <0.001* | 0.66 (0.548, 0.795) | <0.001* | 0.719 (0.594, 0.871) | <0.001* |
| Exercise frequency ≥3 times/week | 1.058 (0.911, 1.228) | 0.463 | 1.026 (0.882, 1.194) | 0.741 | 0.8 (0.63, 1.015) | 0.066 | 0.749 (0.587, 0.956) | 0.020* |
| Weekly exercise duration ≥150min/week | 0.841 (0.763, 0.927) | <0.001* | 0.845 (0.765, 0.932) | <0.001* | 0.715 (0.606, 0.842) | <0.001* | 0.721 (0.61, 0.852) | <0.001* |

Non-proliferative diabetic retinopathy (NPDR), Odds ratio (OR), confidence interval (CI); [a]compared with mild NPDR patients; [b]compared with moderate NPDR patients; * $p<0.05$, a significantly influencing factor in the logistics regression equation.

The unadjusted model is a univariate logistic regression, while the adjusted model is a multivariate logistic regression that has been adjusted for age, sex, duration of diabetes, overweight, control of blood pressure, glycaemic control, insulin use, smoking, alcohol consumption, arterio-venous ratio, and peripheral arterio-venous ratio

## Table S5. Differences in the characteristics of patients with type 2 diabetes between the active and inactive groups (n = 3,669)

| | Inactive (n=3062) | Active (n=607) | T /$\chi^2$ | P-value |
|---|---|---|---|---|
| Age (year, mean±SD) * | 63.2 ± 6.57 | 62.59 ± 6.46 | 2.08 | 0.037* |
| Duration of diabetes (year, mean±SD) | 7.55 ± 5.29 | 7.13 ± 5.44 | 1.76 | 0.078 |
| Body mass index (kg/m$^2$, mean±SD) * | 24.5 ± 2.91 | 24.95 ± 3.12 | -3.46* | <0.001* |
| Haemoglobin A1c (%, mean±SD) | 7.25 ± 1.11 | 7.24 ± 1.09 | 0.20 | 0.840 |
| Mean arterial pressure (mmHg, mean±SD) * | 95.1 ± 4.2 | 96 ± 4.82 | -4.73* | <0.001* |
| Exercise frequency (times/week, mean±SD) * | 4.35 ± 2.3 | 5.84 ± 2.31 | -14.56* | <0.001* |
| Time per exercise (min, mean±SD) * | 223.28 ± 214.77 | 355.92 ± 260.86 | -13.38* | <0.001* |
| CRAE_2017 (μm, mean±SD) | 139.09 ± 14.75 | 138.49 ± 15.71 | 0.91 | 0.361 |
| CRVE_2017 (μm, mean±SD) | 229.11 ± 25.22 | 229 ± 24.36 | 0.1 | 0.921 |
| AVR_2017(mean±SD) | 0.61 ± 0.07 | 0.61 ± 0.07 | 0.85 | 0.394 |
| PRAE_2017 (μm, mean±SD) | 137.6 ± 15.43 | 136.94 ± 16.65 | 0.94 | 0.345 |
| PRVE_2017 (μm, mean±SD) | 225.09 ± 26.96 | 225.44 ± 25.81 | -0.3 | 0.768 |
| PAVR_2017(mean±SD) | 0.62 ± 0.07 | 0.61 ± 0.07 | 1.72 | 0.085 |
| △CRAE (mean±SD) | -0.31 ± 9.68 | 0.22 ± 10.46 | -1.21 | 0.225 |
| △CRVE (mean±SD) | -1.07 ± 9.74 | -1.04 ± 10.48 | -0.08 | 0.935 |
| △AVR (mean±SD) | 1.38 ± 11.01 | 1.94 ± 11.84 | -1.13 | 0.256 |
| △PRAE (mean±SD)* | -0.83 ± 10.85 | 0.33 ± 12.01 | -2.36* | 0.018* |
| △PRVE (mean±SD) | -1.18 ± 11.62 | -1.29 ± 11.19 | 0.22 | 0.823 |
| △PAVR (mean±SD)* | 1.12 ± 11.73 | 2.23 ± 11.56 | -2.15* | 0.032* |
| Diabetic retinopathy (yes) * | 23.45% | 18.95% | 5.85* | 0.017* |
| Sex (female) * | 59.18% | 49.59% | 19.07* | <0.001* |
| Overweight (yes) | 69.37% | 72.82% | 2.87 | 0.099 |
| Control of glucose (accordant) | 41.9% | 42.01% | 0.00 | 0.964 |
| Control of blood pressure (accordant) * | 45.89% | 37.56% | 14.21* | <0.001* |
| Smoke (yes,) * | 88.5% | 83.36% | 12.38* | <0.001* |
| Drink (yes) * | 95.46% | 92.75% | 7.85* | 0.008* |
| Insulin (used) | 9.7% | 10.05% | 0.07 | 0.765 |

Central retinal artery equivalent (CRAE), central retinal vein equivalent (CRVE), arterio-venous ratio (AVR), peripheral retinal artery equivalent (PRAE), peripheral retinal vein equivalent (PRVE), and peripheral arterio-venous ratio (PAVR); independent t-test was used for continuous variables and chi-square test was used for categorical variables; *$p$<0.05, significant difference between patients with and without active physical activity.

Age, sex, and duration of diabetes refer to baseline values, whereas anthropometric and PA data are expressed as 3-year averages from 2017 to 2020.

## Table S6. Effect of physical activity on the incidence of DR

| | No (Number, %) | Yes (Number, %) | $\chi^2$ | P-value |
|---|---|---|---|---|
| Active physical activity* | 718, 23.45% | 115, 18.95% | 6.47* | 0.011* |
| Moderate/high exercise intensity* | 666, 23.73% | 167, 19.37% | 8.49* | 0.004* |
| Exercise frequency ≥ 3 times/week | 146, 24.58% | 687, 22.34% | 1.17 | 0.279 |
| Weekly exercise duration ≥ 150 min/week | 311, 23.19% | 522, 22.42% | 0.50 | 0.481 |

Diabetic retinopathy (DR); $\chi^2$ test was used; *$p<0.05$, significantly different between the two groups

**Table S7. The 19 single nucleotide polymorphism loci ultimately included in the two-sample Mendelian randomization analysis of n**

| | SNP | EA.exp | OA.exp | EA.out | OA.out | beta.exp | beta.out | eaf.exp | eaf.out | remove | palindromi | ambiguous | id.out | chr.out |
|---|---|---|---|---|---|---|---|---|---|---|---|---|---|---|
| 1 | rs1014049 | T | C | T | C | -0.01178 | 0.005292 | 0.388987 | 0.451567 | FALSE | FALSE | FALSE | JEUPI9 | 9 |
| 2 | rs10868649 | T | G | T | G | -0.01401 | -0.00362 | 0.20109 | 0.226574 | FALSE | FALSE | FALSE | JEUPI9 | 9 |
| 3 | rs11106862 | A | G | A | G | -0.01188 | 0.008521 | 0.554594 | 0.52119 | FALSE | FALSE | FALSE | JEUPI9 | 12 |
| 4 | rs11158809 | C | A | C | A | -0.01122 | 0.033101 | 0.432667 | 0.428548 | FALSE | FALSE | FALSE | JEUPI9 | 14 |
| 5 | rs1158619 | C | A | C | A | -0.01268 | 0.004649 | 0.729861 | 0.632982 | FALSE | FALSE | FALSE | JEUPI9 | 6 |
| 6 | rs116980906 | T | C | T | C | -0.02512 | -0.01438 | 0.063744 | 0.058314 | FALSE | FALSE | FALSE | JEUPI9 | 8 |
| 7 | rs11748674 | G | A | G | A | 0.028953 | -0.01603 | 0.038415 | 0.089254 | FALSE | FALSE | FALSE | JEUPI9 | 5 |
| 8 | rs13272557 | G | A | G | A | -0.01543 | 0.038463 | 0.17042 | 0.209161 | FALSE | FALSE | FALSE | JEUPI9 | 8 |
| 9 | rs1375524 | A | G | A | G | -0.01165 | 0.01127 | 0.381727 | 0.398349 | FALSE | FALSE | FALSE | JEUPI9 | 3 |
| 10 | rs145442366 | A | G | A | G | -0.0284 | 0.014108 | 0.039343 | 0.018003 | FALSE | FALSE | FALSE | JEUPI9 | 5 |
| 11 | rs1461583 | G | A | G | A | 0.013542 | -0.01337 | 0.205849 | 0.152494 | FALSE | FALSE | FALSE | JEUPI9 | 14 |
| 12 | rs17829387 | T | C | T | C | 0.070644 | -0.03737 | 0.007662 | 0.011424 | FALSE | FALSE | FALSE | JEUPI9 | 4 |
| 13 | rs183569814 | T | C | T | C | -0.04887 | -0.09075 | 0.015955 | 0.012822 | FALSE | FALSE | FALSE | JEUPI9 | 4 |
| 14 | rs203482 | A | G | A | G | 0.011138 | -0.02645 | 0.398733 | 0.344915 | FALSE | FALSE | FALSE | JEUPI9 | 17 |
| 15 | rs2726527 | A | G | A | G | 0.012263 | 0.000704 | 0.364469 | 0.313426 | FALSE | FALSE | FALSE | JEUPI9 | 4 |
| 16 | rs3001723 | A | G | A | G | 0.012731 | -0.01831 | 0.299804 | 0.257256 | FALSE | FALSE | FALSE | JEUPI9 | 1 |
| 17 | rs41292143 | T | C | T | C | -0.03949 | 0.081533 | 0.022604 | 0.004836 | FALSE | FALSE | FALSE | JEUPI9 | 1 |
| 18 | rs74582032 | A | G | A | G | -0.0318 | 0.075781 | 0.033348 | 0.032444 | FALSE | FALSE | FALSE | JEUPI9 | 7 |
| 19 | rs9489931 | G | A | G | A | 0.012074 | -0.00618 | 0.44031 | 0.493547 | FALSE | FALSE | FALSE | JEUPI9 | 6 |

SNP, Single Nucleotide Polymorphism; EA, effect allele; OA, other allele

**ıoderate-intensity activity and diabetic retinopathy**

| pos.out | pval.out | se.out | outcome | mr_keep.c | pval_origir | samplesiz | pval.exp | chr.exp | se.exp |
|---|---|---|---|---|---|---|---|---|---|
| 27228505 | 0.699861 | 0.013728 | finngen_R12_DM_RETINOPATHY_EXMORE | TRUE | reported | 251031 | 1.19E-06 | 9 | 0.002425 |
| 70168349 | 0.825208 | 0.016371 | finngen_R12_DM_RETINOPATHY_EXMORE | TRUE | reported | 251031 | 2.82E-06 | 9 | 0.002991 |
| 93120641 | 0.533714 | 0.013692 | finngen_R12_DM_RETINOPATHY_EXMORE | TRUE | reported | 251031 | 8.07E-07 | 12 | 0.002408 |
| 69582408 | 0.016748 | 0.013837 | finngen_R12_DM_RETINOPATHY_EXMORE | TRUE | reported | 251031 | 4.17E-06 | 14 | 0.002439 |
| 159872119 | 0.74037 | 0.014031 | finngen_R12_DM_RETINOPATHY_EXMORE | TRUE | reported | 251031 | 2.18E-06 | 6 | 0.002676 |
| 38632020 | 0.624991 | 0.029429 | finngen_R12_DM_RETINOPATHY_EXMORE | TRUE | reported | 251031 | 2.11E-07 | 8 | 0.00484 |
| 12644747 | 0.504998 | 0.024043 | finngen_R12_DM_RETINOPATHY_EXMORE | TRUE | reported | 251031 | 2.36E-06 | 5 | 0.006134 |
| 3282797 | 0.021673 | 0.016752 | finngen_R12_DM_RETINOPATHY_EXMORE | TRUE | reported | 251031 | 1.10E-06 | 8 | 0.003167 |
| 54121968 | 0.41856 | 0.013932 | finngen_R12_DM_RETINOPATHY_EXMORE | TRUE | reported | 251031 | 1.89E-06 | 3 | 0.002446 |
| 64338504 | 0.782492 | 0.051103 | finngen_R12_DM_RETINOPATHY_EXMORE | TRUE | reported | 251031 | 3.67E-06 | 5 | 0.006135 |
| 98171658 | 0.480043 | 0.018933 | finngen_R12_DM_RETINOPATHY_EXMORE | TRUE | reported | 251031 | 4.10E-06 | 14 | 0.00294 |
| 16940797 | 0.565033 | 0.064945 | finngen_R12_DM_RETINOPATHY_EXMORE | TRUE | reported | 251031 | 7.84E-07 | 4 | 0.014302 |
| 184741600 | 0.137886 | 0.061163 | finngen_R12_DM_RETINOPATHY_EXMORE | TRUE | reported | 251031 | 6.96E-07 | 4 | 0.009847 |
| 19923897 | 0.065503 | 0.014361 | finngen_R12_DM_RETINOPATHY_EXMORE | TRUE | reported | 251031 | 4.22E-06 | 17 | 0.002421 |
| 105372023 | 0.961852 | 0.014715 | finngen_R12_DM_RETINOPATHY_EXMORE | TRUE | reported | 251031 | 6.03E-07 | 4 | 0.002457 |
| 43572014 | 0.239537 | 0.01557 | finngen_R12_DM_RETINOPATHY_EXMORE | TRUE | reported | 251031 | 8.28E-07 | 1 | 0.002583 |
| 46192545 | 0.404184 | 0.097741 | finngen_R12_DM_RETINOPATHY_EXMORE | TRUE | reported | 251031 | 1.41E-06 | 1 | 0.008187 |
| 8573408 | 0.05359 | 0.039262 | finngen_R12_DM_RETINOPATHY_EXMORE | TRUE | reported | 251031 | 2.77E-06 | 7 | 0.006784 |
| 97855823 | 0.64684 | 0.013491 | finngen_R12_DM_RETINOPATHY_EXMORE | TRUE | reported | 251031 | 4.69E-07 | 6 | 0.002396 |

| pos.exp | id.exp | exposure | mr_keep.exp | pval_origin.exp | data_source.exp | action | SNP_inde | mr_keep | samplesize.ou | P-value | F-statistics |
|---|---|---|---|---|---|---|---|---|---|---|---|
| 2.7E+07 | ukb-a-509 | \|\| id:ukb-a-509 | TRUE | reported | igd | 2 | 1 | TRUE | 73226 | 9.40E-05 | 23.59026 |
| 7.3E+07 | ukb-a-509 | \|\| id:ukb-a-509 | TRUE | reported | igd | 2 | 1 | TRUE | 73226 | 8.74E-05 | 21.93423 |
| 9.4E+07 | ukb-a-509 | \|\| id:ukb-a-509 | TRUE | reported | igd | 2 | 1 | TRUE | 73226 | 9.70E-05 | 24.34154 |
| 7E+07 | ukb-a-509 | \|\| id:ukb-a-509 | TRUE | reported | igd | 2 | 1 | TRUE | 73226 | 8.44E-05 | 21.18599 |
| 1.6E+08 | ukb-a-509 | \|\| id:ukb-a-509 | TRUE | reported | igd | 2 | 1 | TRUE | 73226 | 8.94E-05 | 22.43338 |
| 3.8E+07 | ukb-a-509 | \|\| id:ukb-a-509 | TRUE | reported | igd | 2 | 1 | TRUE | 73226 | 0.000107 | 26.93412 |
| 1.3E+07 | ukb-a-509 | \|\| id:ukb-a-509 | TRUE | reported | igd | 2 | 1 | TRUE | 73226 | 8.87E-05 | 22.27633 |
| 3140319 | ukb-a-509 | \|\| id:ukb-a-509 | TRUE | reported | igd | 2 | 1 | TRUE | 73226 | 9.46E-05 | 23.73947 |
| 5.4E+07 | ukb-a-509 | \|\| id:ukb-a-509 | TRUE | reported | igd | 2 | 1 | TRUE | 73226 | 9.04E-05 | 22.70221 |
| 6.4E+07 | ukb-a-509 | \|\| id:ukb-a-509 | TRUE | reported | igd | 2 | 1 | TRUE | 73226 | 8.54E-05 | 21.43057 |
| 9.9E+07 | ukb-a-509 | \|\| id:ukb-a-509 | TRUE | reported | igd | 2 | 1 | TRUE | 73226 | 8.45E-05 | 21.21932 |
| 1.7E+07 | ukb-a-509 | \|\| id:ukb-a-509 | TRUE | reported | igd | 2 | 1 | TRUE | 73226 | 9.72E-05 | 24.39817 |
| 1.9E+08 | ukb-a-509 | \|\| id:ukb-a-509 | TRUE | reported | igd | 2 | 1 | TRUE | 73226 | 9.81E-05 | 24.62638 |
| 2E+07 | ukb-a-509 | \|\| id:ukb-a-509 | TRUE | reported | igd | 2 | 1 | TRUE | 73226 | 8.43E-05 | 21.16361 |
| 1.1E+08 | ukb-a-509 | \|\| id:ukb-a-509 | TRUE | reported | igd | 2 | 1 | TRUE | 73226 | 9.92E-05 | 24.90446 |
| 4.4E+07 | ukb-a-509 | \|\| id:ukb-a-509 | TRUE | reported | igd | 2 | 1 | TRUE | 73226 | 9.68E-05 | 24.29194 |
| 4.7E+07 | ukb-a-509 | \|\| id:ukb-a-509 | TRUE | reported | igd | 2 | 1 | TRUE | 73226 | 9.27E-05 | 23.26707 |
| 8613038 | ukb-a-509 | \|\| id:ukb-a-509 | TRUE | reported | igd | 2 | 1 | TRUE | 73226 | 8.75E-05 | 21.97386 |
| 9.8E+07 | ukb-a-509 | \|\| id:ukb-a-509 | TRUE | reported | igd | 2 | 1 | TRUE | 73226 | 0.000101 | 25.39005 |

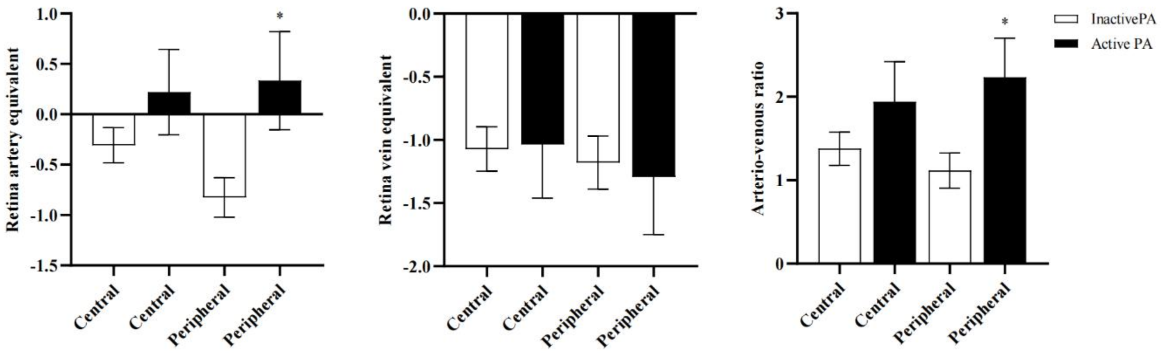


**Figure S2. Differences in retinal vascular parameters changes between the active and inactive groups**

(A) Retina artery equivalent
(B) Retina vein equivalent
(C) Arterio-venous ratio
Physical activity (PA); *$p$<0.05, significantly difference between the active and inactive groups.
